# Physical Properties, Spectroscopic, Microscopic, X-ray, and Chemometric Analysis of Starch Films Enriched with Selected Functional Additives

**DOI:** 10.3390/ma14102673

**Published:** 2021-05-20

**Authors:** Maciej Combrzyński, Tomasz Oniszczuk, Karol Kupryaniuk, Agnieszka Wójtowicz, Marcin Mitrus, Marek Milanowski, Jakub Soja, Iwona Budziak-Wieczorek, Dariusz Karcz, Daniel Kamiński, Sławomir Kulesza, Karolina Wojtunik-Kulesza, Kamila Kasprzak-Drozd, Marek Gancarz, Iwona Kowalska, Lidia Ślusarczyk, Arkadiusz Matwijczuk

**Affiliations:** 1 Department of Thermal Technology and Food Process Engineering, University of Life Sciences in Lublin, Głęboka 31, 20-612 Lublin, Poland; tomasz.oniszczuk@up.lublin.pl (T.O.); agnieszka.wojtowicz@up.lublin.pl (A.W.); marcin.mitrus@up.lublin.pl (M.M.); marek.milanowski@up.lublin.pl (M.M.); jakubsoja97@wp.pl (J.S.); 2 Department of Chemistry, University of Life Sciences in Lublin, 20-950 Lublin, Poland; iwona.budziak@up.lublin.pl; 3 Department of Chemical Technology and Environmental Analytics (C1), Faculty of Chemical Engineering and Technology, Cracow University of Technology, Warszawska 24, 31-155 Kraków, Poland; dariusz.karcz@pk.edu.pl; 4 Department of General and Coordination Chemistry and Crystallography, Institute of Chemical Sciences, Maria Curie-Skłodowska University in Lublin, pl. Marii Curie-Skłodowskiej 2, 20-031 Lublin, Poland; daniel.kaminski@umcs.pl; 5 Department of Mechatronics, Faculty of Technical Sciences, University of Warmia and Mazury in Olsztyn, Oczapowskiego 11, 10-710 Olsztyn, Poland; slawek.kulesza@gmail.com; 6 Department of Inorganic Chemistry, Medical University in Lublin, 20-059 Lublin, Poland; k.wojtunik@o2.pl (K.W.-K.); kamilakasprzakdrozd@gmail.com (K.K.-D.); 7 Institute of Agrophysics Polish Academy of Sciences, Doświadczalna 4, 20-290 Lublin, Poland; m.gancarz@ipan.lublin.pl; 8 Department of Biochemistry and Crop Quality, Institute of Soil Science and Plant Cultivation, State Research Institute, 24-100 Puławy, Poland; ikowalska@iung.pulawy.pl; 9 Department of Biophysics, University of Life Sciences in Lublin, Akademicka 13, 20-950 Lublin, Poland; lidia.slusarczyk@up.lublin.pl

**Keywords:** thermoplastic starch with functional additives, extrusion-cooking, biopolymer films, molecular spectroscopy and chemometric analysis, AFM, nanomechanical mapping and X-ray

## Abstract

Biodegradable materials are used in the manufacture of packaging and compostable films and various types of medical products. They have demonstrated a large number of potential practical applications in medicine and particularly in the treatment of various cardiac, vascular, and orthopedic conditions in adults as well in children. In our research, the extrusion-cooking technique was applied to prepare thermoplastic starch (TPS), which was then utilized to obtain environmentally friendly starch-based films. Potato starch was the basic raw material exploited. Polyvinyl alcohol and keratin were used as functional additives in amounts from 0.5 to 3%, while 20% of glycerol was harnessed as a plasticizer. The processing of the thermoplastic starch employed a single screw extruder-cooker with an L/D ratio of 16. The film blowing process was carried out using a film-blowing laboratory line with L/D = 36. FTIR Spectroscopy was applied for the assignment of the prominent functional groups. The results showed that the processing efficiency of thermoplastic starch with functional additives varied depending on the level of polyvinyl alcohol and keratin addition. Moreover, the FTIR data correlated with the changes in the physical properties of the tested films. The analysis of FTIR spectra revealed several changes in the intensity of bands originating from stretching vibrations characteristic of the –OH substituent. The changes observed depended on the presence/lack of the hydrogen bonding occurring upon interactions between the starch molecules and the various additives used. In addition, notable changes were observed in bands assigned to glycoside bonds in the starch.

## 1. Introduction

The bioplastics market, which started to emerge not even 20 years ago, is growing rapidly and has already reached impressive production volumes. It is estimated that the global production of plastics reached over 400 million tons in 2015. In 2018, the global production of plastics totaled 359 million tons [1]. Despite the economic slowdown of 2009, which seriously undermined the chemical and plastics industries, demand for bioplastics was on the rise, both in Europe (from 5% to 10%), as well as in North America and Asia. In 2017, it was estimated that the global bioplastics manufacturing capacity would increase from 2.05 million tons to approx. 2.44 million tons by 2022. In a 2009 report, European Bioplastics envisaged the growth of the biopolymer market by an average of 19% year-on-year between 2007 and 2020. The use of polymers, such as PLA (polylactic acid) and PHAs (polyhydroxyalkanoates), has the greatest impact on the ever-higher supply of biodegradable plastics. The largest market for biodegradable polymers is Europe. Within the European continent, the greatest developments in biopolymers are seen in Germany, Austria, Benelux, and Scandinavia [2].

Biocomposite materials, notably biodegradable and bio-based, are practically absent on the Polish market. Their potential has not been recognized and, above all, not used. The reasons behind this are: the low worldwide spread of biopolymer composites, lack of adequate information on possible applications, presuppositions concerning the processing, and the unsatisfactory properties of these materials. The large production and consumption figures in Europe are mainly attributable to the legislative setting, yet it is the United States that has the largest production capacity. This is thanks to the PLA manufacturing facility run by NatureWorks LLC, although not exploited to its full capacity. Given the impressive development in technology in recent years and the competitiveness of bioplastics, the range of products that can be made from or with biocomposites is already remarkable and new opportunities are already around the corner. Biopolymer composite materials can be used mainly as products with a short life cycle, for example, packaging, containers, buckets, boxes, films for mulching, biodegradable pots, products for the catering industry, etc. What is more, biopolymers with high mechanical properties and extended useful life can be used as a cover for composites used as construction materials [3].

Such polymers provide a reasonable alternative to traditional synthetic materials, the disposal of which harms the natural environment. Packaging and packaging waste is governed by a law (Journal of Laws of 2013, item 888, as amended, and Directive (EU) 2018/851 of the European Parliament and of the Council) that demands that certain levels of waste be subjected to recovery, including recycling. Biocomposites are composite materials with at least one “bio” component. Therefore, biocomposites are also petrochemical polymers, biopolymers filled with natural fibers, and biopolymers with synthetic fibers. One group of biodegradable bio-based polymers are those produced from plants, among them are polysaccharides obtained by fractionation, for example, starch obtained from potatoes, maize, rice, or wheat [4].

The production of starch polymers begins with the separation of starch from the raw material. To obtain thermoplastic starch, the crystalline structure of its grains is destroyed by extrusion with the addition of a plasticizer (e.g., glycerol). Thermoplastic starch (TPS) often contains more than 70% fully biodegradable starch. This material exhibits moderate mechanical properties. Consequently, most starch-based polymers are thermoplastic mixtures of starch with other polymers. Starch polymers have also been proven to be easy to process. They can be made into end products by using slightly modified standard thermoplastic processing equipment, mainly by extrusion, injection, hot forming, blowing, or foaming. This material, however, has certain disadvantages, one of them being reduced resistance to solvents, especially water [5]. Hence, of great importance is the use of appropriate functional additives that alter the properties of pure thermoplastic starch. Besides functional additives based on petrochemicals, there are also natural and biodegradable materials that, when used in a reasonable manner, can add certain desired characteristics to any final product [6,7]. These are, for example, polyvinyl alcohol and keratin [8,9].

The aim of the research was an in-depth analysis of both the thermoplastic starch extrusion process and the physicochemical, spectroscopic, and surface properties, as well as the internal structure of new types of biodegradable films with various functional additives. For this purpose, the following were engaged: infrared spectroscopy (Fourier-transform infrared spectroscopy, FTIR) combined with PCA (principal component analysis) and HCA (hierarchical clustering analysis), X-ray diffraction (XRD analysis), and detailed surface studies employing AFM (atomic force microscopy).

The final surface texture carries a unique imprint of complex processes occurring simultaneously in the bulk and at the interfacial boundaries of solids. Hence, in-depth, multi-aspect characterization of the surface needs to be carried out to assess the effective exploitation of the functional properties of novel materials. Atomic force microscopy (AFM) proved to be an extremely useful and accurate method in that matter according to results reported in such fields as metallurgy and ceramics [9,10,11], biology and medicine [12,13,14], environmental studies [15], and others. This technique was also successfully applied to polymers, for example, to study temperature-induced changes in the surface topography of low-density polyethylene (LDPE) [16]. To our best knowledge, there are no studies concerning such a form of properties evaluation of biopolymers based on potato starch.

## 2. Materials and Methods

Potato starch of the Superior Standard type (PEPEES S. A., Łomża, Poland) was used in the study. The moisture content of the starch was 15.6% and the pH was 7.2. Polyvinyl alcohol PVA (Avantor-POCH S.A., Gliwice, Poland) was assessed for employment as a functional additive in the amount of 0.5, 1.0, 2.0, and 3.0% (*w*/*w*). PVA. This has a melting temperature of 160–200 °C and is an odor-free white powder with a density of 1.2–1.3 g·cm^−3^. The application of Keratin (Noble Health, Radom, Poland) was also established as a functional additive in the amount of 0.5, 1.0, and 1.5% (*w*/*w*). During bio-granulate production, a plasticizer-glycerol of 99.5% purity (Brenntag Polska sp. z o.o., Kędzierzyn-Koźle, Poland) was added in the amount of 20% (*w*/*w*).

In order to homogenize each blend, the samples (shown in Table 1) were left in sealed plastic bags for 24 h. Before the extrusion-cooking process began, the raw material blends were re-mixed.

### 2.1. Process of Obtaining Starch-Based Film

**Figure 1:**
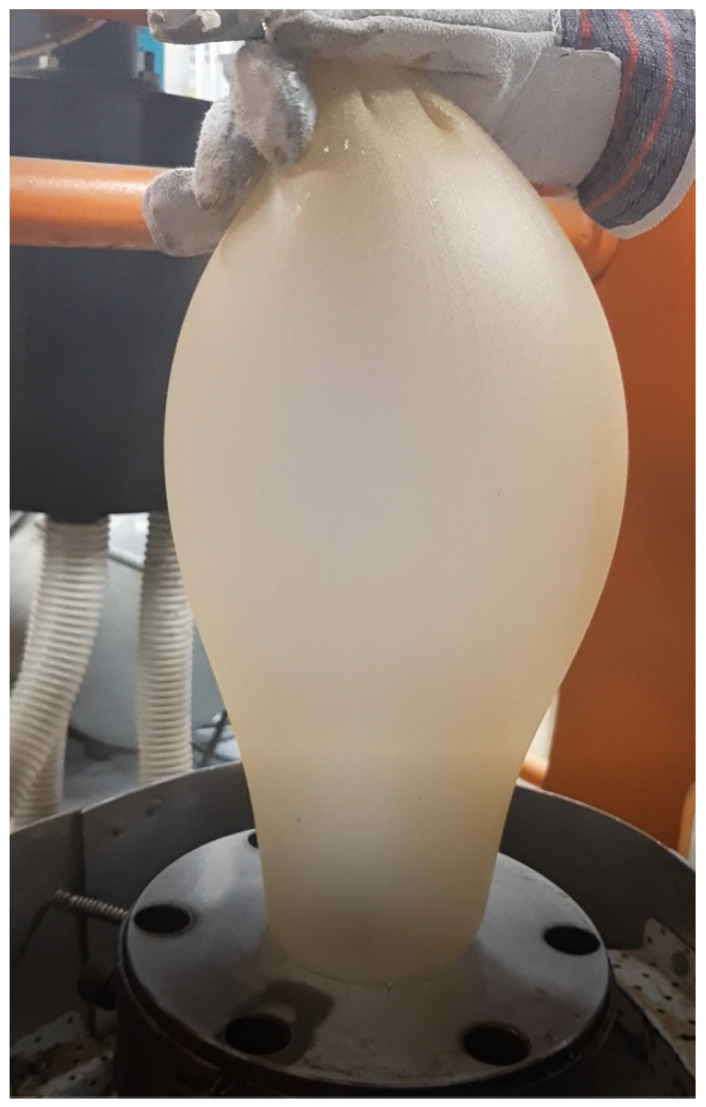
Starch-based film with functional substances blowing in laboratory.

The starch-based films (Figure 1) were produced using a two-step process:

Step 1: Biopolymer granulates of TPS were obtained using extrusion-cooking equipment—a modified single screw extrusion-cooker TS-45 (ZMCh Metalchem Gliwice, Poland). The TPS was processed with a screw speed of 100 rpm and with a circular forming die with 3 mm of diameter. Extrusion-cooking was carried out at a temperature range from 60 to 100 °C. The product was shaped using a high-speed knife mounted at the head. Its rotational speed was adapted to the desired dimensions of the extrudates, that is, a diameter of approx. 3 mm. The product obtained in the process was stabilized at ambient temperature in a shelf dryer. After cooling, the extrudates were stored in bags and sampled for further tests.

**Figure 2:**
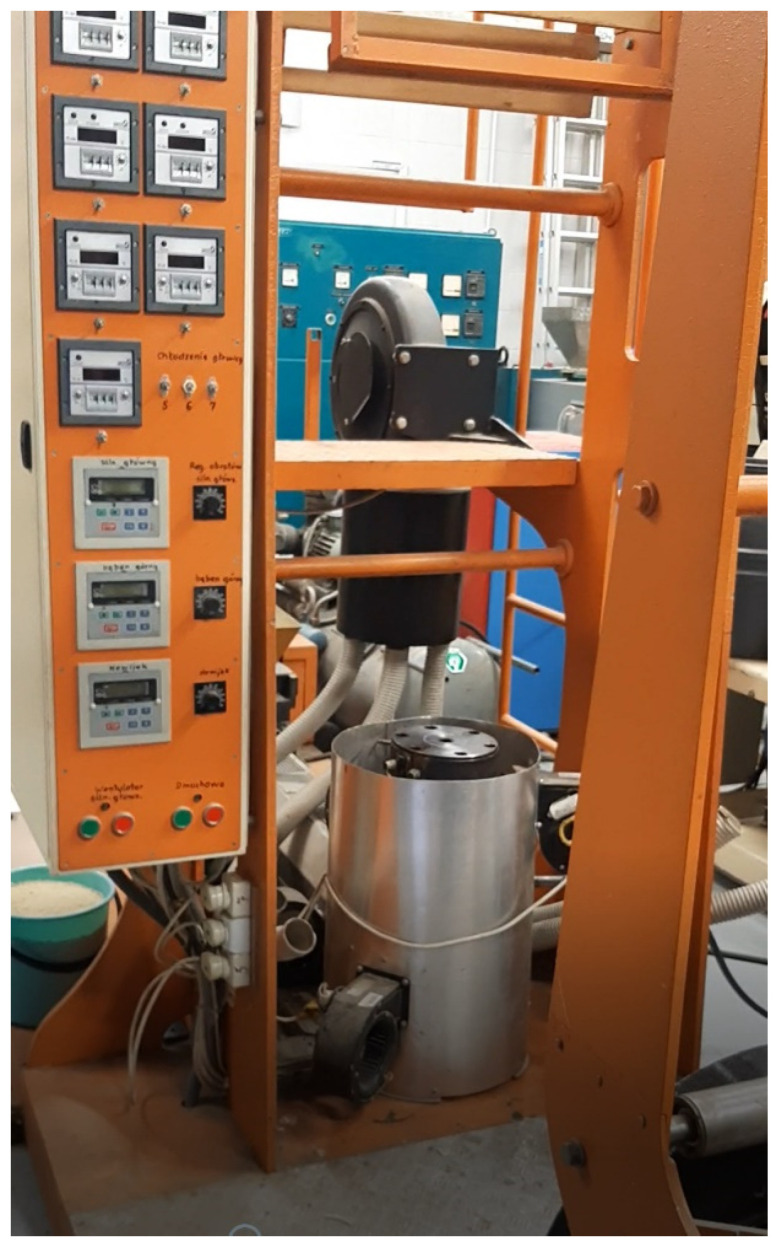
Film-blowing laboratory line.

Step 2: Starch-based films were generated from the above-mentioned granulates using a film-blowing laboratory line produced by SAVO Ltd. Co., Warsaw, Poland (Figure 2). The film blowing process was carried out with a plastic extruder with L/D = 36, and with a screw speed from 30 to 100 rpm. During extrusion, a die-mold with a nozzle diameter of 80 mm and a working slit of 0.6 mm was used.

For the extrusion-cooking process, drive parameters (including load) were taken into account, as well as the efficiency of extrusion-cooking for the individual raw material blends. The efficiency of extrusion-cooking of starch-based materials was determined by the sampling of extrudates at a specific time. The efficiency was calculated according to the formula [17].

(1) $$Q=\frac{m}{t}\;\left({\mathrm{kg}\;h}^{-1}\right)$$

where:

Q: process efficiency (kg h^−1^)

m: mass of extrudate obtained in the measurement (kg),

t: time of measurement (h).

Energy intensity was determined based on the specific mechanical energy (SME) value, by exploiting the formula proposed by Ryu and Ng [18].

(2) $$\mathrm{SME}=\frac{n\;P\;O}{n_{m}\;Q}\;\left({\mathrm{kWh}\;\mathrm{kg}}^{-1}\;\right)$$

where:

SME: Specific Mechanical Energy (kWh kg^−1^)

n: extruder-cooker screw speed (1 s^−1^),

n_m_: extruder-cooker nominal speed (1 s^−1^),

O: drive load v. maximum load (%),

P: nominal power (kW),

Q: extrusion-cooking efficiency (kg h^−1^).

### 2.2. AFM Nanomechanical Mapping

Maps of the surface properties of the extruded biopolymer films were examined on both sides of the samples by means of the application of the Atomic Force Microscopy method (AFM), using a Multimode 8 instrument (Bruker, Ettlingen, Germany). The measurements were carried out in tapping mode, as this provided direct control of the tip–sample interaction so that the maximum repulsive force did not exceed 500 pN. Each image covered a 1 × 1 µm^2^ scan area, was taken as a 256 × 256 step, and corresponded to a lateral resolution slightly below 4 nm. Apart from the topography, however, captured data were also helpful in studying variations in the pseudo-Young’s modulus and tip–surface adhesion force that both contributed to the nanomechanical properties of the surface. AFM measurement was done by employing a silicon-scanning probe (TAP150GD-G, BudgetSensors, Sofia, Bulgaria) with a nominal spring constant of 5 N/m, resonance frequency of 150 kHz, and tip radius of 10 nm. Prior to any analysis, height images were plane-fitted in order to remove surface tilt and then flattened row by row to level the neighboring lines in the image.

### 2.3. FTIR Spectroscopy

The ATR-FTIR spectra were recorded within the range of 4000 and 400 cm^−1^ with the use of a Vector 3300 spectrometer (Bruker Optik GmbH, Ettlingen, Germany). The measurements were carried out in solvents at a temperature of 23 °C using a ZnSe trough (45° cut, yielding 10 internal reflections) crystal plate for liquids and were background-corrected [19,20].

## 3. Multivariate Analysis

### 3.1. Principal Component Analysis (PCA)

Principal component analysis (PCA) is one of the most commonly utilized statistical data reduction methods. The purpose of this method is to decompose the original set of variables into a new set of uncorrelated variables called principal components (PCs), that retain as much of the information in the original variables as possible [21,22]. The general PCA model can be expressed as:

(3) $$X={\mathrm{TP}}^{T}+E$$

where X is the data matrix to be analyzed, T is called the score matrix, P is the loading matrix, and E is the residual, respectively.

### 3.2. Hierarchical Clustering Analysis (HCA)

Another applicable chemometrics method is hierarchical clustering analysis [23,24]. HCA is an exploratory data analysis that is used for detecting similarities in the variables set and for classifying them into clusters. Graphical representation of results is a tree-based representation of the objects, deemed a “dendrogram”.

Multivariate analyses, including principal component analysis (PCA) and hierarchical cluster analysis (HCA), were performed for the FTIR spectra. Grams/AI 8.0 software (Thermo Scientific, Waltham, MA, USA) was applied for multi-point baseline correction, Savitzky–Golay smoothing, as well as Y offset correlation, and points were set to zero prior to the analysis. The PCA and HCA analyses were performed within the range of 3700–600 cm^−1^ and 1800–600 cm^−1^. In the hierarchical cluster analysis, the Ward method and average linkage distance between the pairs of samples were used as linkage criteria. Pearson correlation distance between the pairs of samples was assessed as a distance measure. Statistica 13 software (TIBCO Software Inc., Palo Alto, CA, USA) was applied for chemometrics analysis. Samples were allocated into six groups in relation to Table 1.

### 3.3. XRD Analysis

Films that were 1 mm thick with dimensions of 10 × 10 mm were analyzed by applying the powder X-ray diffraction technique, by employing an Empyrean (PANalytical, Warsaw, Poland) diffractometer with a Cu anode as a source for CuKα X-ray radiation (λ = 1.5406 Å). This instrument was equipped with a PIXcel3D detector. All samples were measured over a 2θ range of 4 to 43.5°, with a step size of 0.26° and an exposure time per step of 12.6 s. Diffraction data were assessed utilizing WAXFIT [25] software to determine the degree of crystallinity. All data were normalized, background-corrected (hyperbolic function), and fitted to a model containing 15 Gauss–Cauchy functions that approximate the crystalline phase and 1 function that approximates the amorphous phase. The degree of crystallinity was presented as a ratio between surface areas under the curves of the crystalline phase to the sum of the crystalline and amorphous phases.

## 4. Results and Discussion

### 4.1. Results and Discussion of Extrusion-Cooking Processing

Table 2 provides a listing of the measured results for the efficiency and the energy consumption of the extrusion-cooking process of potato starch with the addition of functional substances. With most of the blends applied, the process was exceptionally constant and free from disturbances that would trigger changes in the energy consumption of the process.

During the manufacturing, it was observed that an increase in polyvinyl alcohol and keratin content resulted in increased efficiency of extrusion-cooking. Higher values were also obtained for samples containing the two functional additives. The highest efficiency was reported for a mixture of starch with the addition of 1.5% of both polyvinyl alcohol and keratin (31.8 kg h^−1^). The observed relationships resemble the results achieved during the extrusion-cooking of thermoplastic starch with functional additives, including PVA [26]. The results also confirm that the efficiency of the extrusion-cooking improved along with the amount of the additive.

When polyvinyl alcohol was added, higher SME was observed as the amount of PVA in the blend was raised. This result is consistent with what other researchers have observed [27]. In contrast, the opposite relationship was reported when PVA was combined with keratin. With an increasing amount of polyvinyl alcohol and keratin, the SME of the extrusion-cooking process decreased. Lower energy consumption values of extrusion-cooking were noted for samples containing both functional additives. The lowest energy consumption was reported for a mixture of starch with the addition of 1.5% of both polyvinyl alcohol and keratin (0.08 kWh kg^−1^). It is worth noting that with these processed blends, the process was extremely stable and constant, which is also evidenced by load tests of the plasticizing system in relation to the extruder-cooker drive.

### 4.2. Results and Discussion of AFM Nanomechanical Imaging

**Figure 3:**
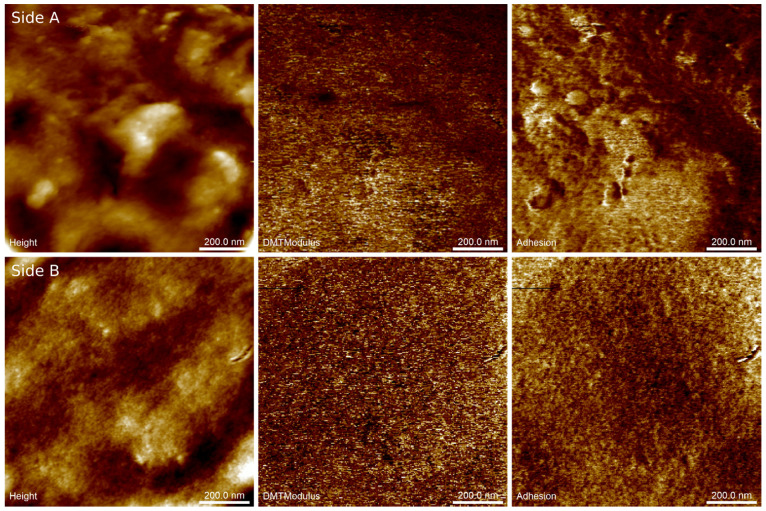
Example results of AFM nanomechanical mapping of both sides of the SGAK-I polymer foil: surface topography (Height), pseudo-Young’s modulus (DMTModulus), tip–surface adhesion force (Adhesion). Scan area is 1 × 1 µm^2^. Side (**A**) internal structure of foil; Side (**B**) external structure of foil.

Figure 3 shows example AFM images of surfaces on both sides of the biopolymer thin foil made with a minor addition of keratin (SGAK-I). The presented images reveal differences in the specific size of dominant structural patterns likely caused by differences in the cooling rate of the material on both sides of the foil. Surface A exhibits the internal structure of the foil during film blowing, which was cooled down with pressured air from a compressor, while surface B shows the external structure coming out from the forming die, as shown in Figure 1. The surface of side A exhibits significant waviness because of the irregular airflow during blowing and a faster cooling rate was then observed at the external surface. Here, the horizontal period of the height variations approaches half a micrometer and is disturbed by randomly placed irregular particles around two orders of magnitude smaller. Side B was found to be significantly flatter, and slight fluctuations of the surface heights occur within the range of single nanometers. Similar observations can be made for the remaining films in this study.

Basically, elastic properties of the materials are derived on the fly from the force-displacement curves recorded when the periodically modulated probe interacts with the sample surface. Initially, the retract curve is fitted using the Derjaguin–Muller–Toporov (DMT) model [28] to obtain the reduced Young’s modulus. Subsequently, this parameter is converted into Young’s modulus providing that the Poisson’s ratio is known. Unfortunately, the sample Poisson’s ratio is not generally accurately known, hence we refer to the obtained elastic modulus as the pseudo-modulus. Notwithstanding, the maps of this modulus demonstrate the structural homogeneity and phase purity of the material.

Imaging of the adhesion forces reveals subtle differences in tip–surface interactions that reflect variations in the thickness of the capillary layer on the surfaces. At several places on side A (ca. 100 nm in diameter), adhesion forces are substantially increased with respect to the remaining area. Note, however, that they are not spatially correlated with any visible structures in the height image. In contrast, distributions of pseudo-Young’s modulus and adhesion forces on side B of the foil were found to be almost uniform, revealing structural and phase homogeneity.

Table 3 provides the averaged parameters of the topographical and mechanical complexity of the surfaces derived from AFM images of the tested films. Here, the parameters related to surface geometry (surface roughness S_q_, autocorrelation length S_al_, and anisotropy ratio S_tr_) are derived from autocorrelation or probability distribution functions, and therefore they ambiguously discriminate between various surface patterns. Notwithstanding these limitations, the performed calculations yielded results scattered enough to demonstrate the dissimilarity of the spatial characteristics of amplitude and surface patterns on both sides of each foil.

Inconclusive dependencies between the tested features of films presented in Table 3 may be the results of inhomogeneity of mixed raw materials with functional additives before film blowing because the blends were prepared at laboratory conditions, not at an industrial scale.

As a rule, surfaces on side A appear rougher than those on side B according to the roughness parameter S_q_ (mean value 3.5 nm). This effect corresponds to the greater amplitude of large-scale surface patterns. In contrast, surfaces on side B are flatter (mean S_q_ is 2.7 nm), which is mainly due to the lesser height variations of nanosized features and a complete lack of larger structures. The surface roughness was found to be slightly larger in SGA samples than in SGAK (4.6 nm vs. 2.1 nm, respectively) and this outcome demonstrates the flattening effect of keratin addition into the liquid mixture. Keratin might reduce the surface roughness in two ways: by increasing the surface tension in the fluid and/or by decreasing the heat flux transmitted outward, which subsequently slows down the cooling of the polymer.

According to the data listed in Table 3, there is straight dependence between surface roughness and specific autocorrelation decay S_al_. The latter parameter defines arbitrarily the lower limit for the lateral size of predominant surface structures, which is found to be 81 nm vs. 71 nm for sides A and B, respectively. Straight correspondence between the horizontal and vertical sizes of the surface patterns demonstrates their rather uniform three-dimensional scaling.

Similar observations can be made concerning the texture anisotropy ratio S_tr_, which increases with increasing surface roughness and autocorrelation length. On one hand, S_tr_ is a measure of the sensitivity of the surface patterns to the observation angle, but on the other, the inverse of S_tr_ provides the magnification factor for the horizontal size of the dominant surface patterns. Hence, the increasing fraction of large-scale surface patterns on side A corresponds to more isotropic, though rough, surfaces; whereas the increasing amount of nanosized features on side B results in pronounced directional alignments of structural components, that is, the surface layer.

The obtained results of nanomechanical mapping generally agree with previous conclusions drawn from height images. Larger values of Young’s pseudo-modulus are found on sides A than B. This leads to the conclusion that elastic properties are determined by the relative content of surface components of various scale lengths. Similar to hardening, faster cooling results in the increasing contribution of large-scale patterns and a stiffer surface, whereas slower cooling leaves a nano-rough, though softer, surface. Likewise, adhesion forces are found to be larger on surfaces with greater roughness. Such an effect is likely due to the retention ability of the polymer to accumulate the capillary layer within the deepest parts of the surface. Here, the addition of keratin lowers the tip–surface interaction through the reduction of the surface roughness (thinner capillary layer) that was discussed in previous paragraphs.

### 4.3. Results and Discussion of FTIR Spectroscopy

ATR and FTIR are very popular techniques employed for elementary analysis of various types of organic compounds and materials containing, for instance, biodegradable components. These materials include, for example, thermoplastic starch, potato starch, technical glycerol, polyvinyl alcohol (PVA), polylactide (PLA), as well as keratin hydrolyzate, and many others. FTIR is also a useful tool for determining the content of the above compounds in various types of products where content significantly affects functionality. The application of FTIR to establish the content of the compounds in materials exhibiting biodegradable properties is confirmed by the increasing number of cases, including ours, reported in the literature on the subject [27,29]. The usefulness of FTIR for the study of this type of samples is mainly due to the fact that the absorption of moieties, such as the carbonyl group bands, especially that occurring during the intensification of the degradation effects in tested materials, or the content of additives used in the starch fraction of a tested product, increases relatively along with their growing volume. It should also be underlined that starch itself reveals very intense and interesting IR bands as it contains carbohydrates and plant polysaccharides. It consists mainly of glucose units linked by α -glycosidic bonds. There are two main fractions: first, unbranched amylose, which is made up of glucose residues linked together by oxygen atoms by α-1,4-glycosidic bonds. The other component is branched amylopectin with additional α-1,6-glycosidic bonds. The incorporation of the above-listed additives, which produce intense IR bands, also suggests a significant modification of molecular interactions in the obtained group of biodegradable film materials. These facts translate into evident changes in the FTIR spectra of the film selected for the study [27,30].

**Figure 4:**
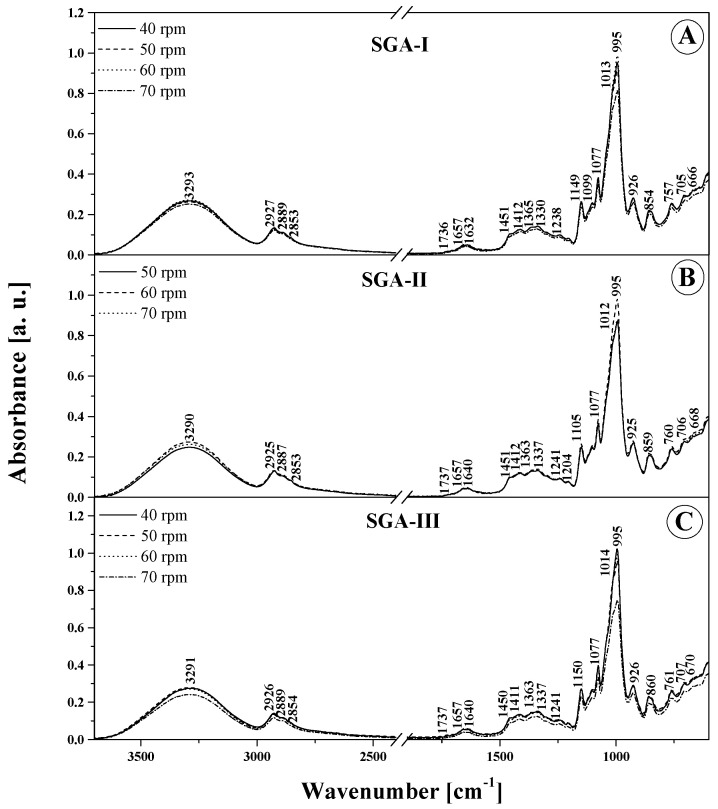
ATR-FTIR absorption spectra for the analyzed films: SGA-I (**A**) and SGA-II (**B**) and SGA-III (**C**). Numbers next to peaks are the wavenumbers for each component. Different lines indicate various screw speeds (rpm) during film blowing processing.

**Figure 5:**
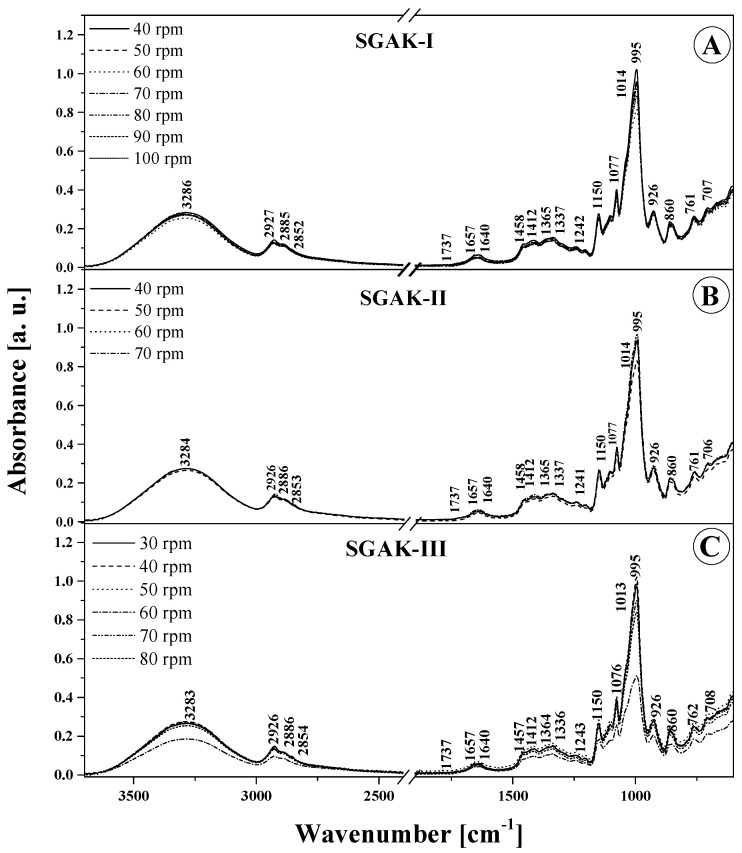
ATR-FTIR absorption spectra for the analyzed films: SGAK-I (**A**), SGAK-II (**B**), and SGAK-III (**C**). Numbers next to peaks are the wavenumbers for each component. Different lines indicate various screw speeds (rpm) during film blowing processing.

For a more convenient analysis of the obtained FTIR spectra, the relevant panels in Figure 4 and Figure 5 demonstrate and Table 4 lists all more significant vibrations, together with their assignment to specific functional groups, based on the available literature [27,31,32,33,34,35]. It is clearly visible that there were noticeable changes to the structure of the tested films after using the selected additives. The changes are also discernible at the molecular level in the form of changes in the IR spectra. This is attributable both to the amount of the additive used and the rotational speed of the extruder device [27,36]. Evidently, all the tested samples contain a low content of water molecules. This confirms the presence of moderately intense bands in the region around 1660 cm^−1^, which should be rated as deformation vibrations –OH, and vibrations in the range of 3550–3100 cm^−1^ with an intense peak at ~3300 cm^−1^. These, in turn, are derived from the stretching vibrations of -OH groups. Both of these vibration regions are also likely to originate from the molecules of the main constituent component of these materials, that is, starch, which contains a significant number of -OH groups. In addition, the additives used also contain -OH groups. The vibrations in the region of 3500–3100 cm^−1^, that is, stretching vibrations of the -OH groups, can also be strengthened by free, internal, and external molecular hydrogen bonds occurring in the starch structure [32,34]. These become particularly active upon the incorporation of the additives that also contain them, and, as the percentage share of selected additives increases, hydrogen bonds may form between them and starch molecules. The bonds modify the starch internal structure and thus alter vibration intensity. It is worth noting that the vibrations from the peak of ~ 1660 cm^−1^, or deformation –OH, can also be strengthened by the vibrations of the water molecules contained within the starch. Another noteworthy vibration range is 3000–2700. These are indicative of the symmetric and asymmetric C–H stretching vibrations in –CH_2_ groups [29]. Moreover, the vibration area from 1200 to 1000 cm^−1^ seems to be relevant as well, with its C–O stretching vibrations. This area exhibits vibrations originating from the C–O–C group naturally occurring in polysaccharides. In contrast, the area from 930 and below down to about 700 cm^−1^ is characteristic of vibrations of the polysaccharide ring, while vibrations falling under this can be ascribed to the pyranose ring in individual units belonging to a single glucose unit.

In the wavenumber range 3550–3100 cm^−1^ (Figure 4 and Figure 5 and Table 4), the characteristic broad band that corresponds to the stretching vibrations of the –OH group displays the highest intensity for both types of SGA and SGAK films for the smallest amounts of additives used and, above all, for the lowest rotational speed values of the extruding device. In these cases, the peak of this spectrum is also slightly shifted towards higher wavenumbers, which implies the formation of more intermolecular hydrogen bonds with water molecules in the samples. This is further evidenced by the greater intensity of the spectrum associated with deformational vibrations of –OH groups at ~1640 cm^−1^, which is associated with vibrations in starch itself. Regarding vibrations in the range of 3100–2700 cm^−1^, typical of –CH_2_ stretching vibrations, the most intense spectra are seen in films with the lowest volumes of additives, especially keratin. This may be attributed to the higher content of amylose and amylopectin, as well as a much greater strength of intermolecular forces in the studied additives than that in alcohol. Moreover, in the FTIR spectra of films modified with all the tested additives, very profound changes were also observed in bands with a peak at around 1240–80 and 940–1200 cm^−1^. These vibrations are related to the skeletal vibrations of the C–O and C–C groups that typify starch and can be reinforced by vibrations originating in the additives [37]. Primarily, this testifies to a very good mixing of ingredients used in the production of film blends, meaning that they are relatively homogeneous. Secondly, it shows the existence of strong interactions between them, as strong hydrogen bonds between individual groups were established. Below about 870 cm^−1^, there are bands characteristic of α -glycosidic bonds, that is, typical of starch. It is also worth highlighting that noticeable changes in this area occur mainly with the increase in the rotational speed of the extruder device. They evidence the structural changes within the glycosidic bond that connects the molecules.

To sum up, it is worth noting that the tested additives modify the FTIR spectrum of the tested films in a noticeable, yet not profound manner. Both for SGA and SGAK films the infrared spectra of the tested materials are very similar over the entire range of additive volumes. This proves the relatively high quality and stability of the material [26]. For a more detailed comparison and in an attempt to identify significant differences in the samples, chemometric analysis was applied further in the work.

## 5. Results of Multivariate Analysis

**Figure 6:**
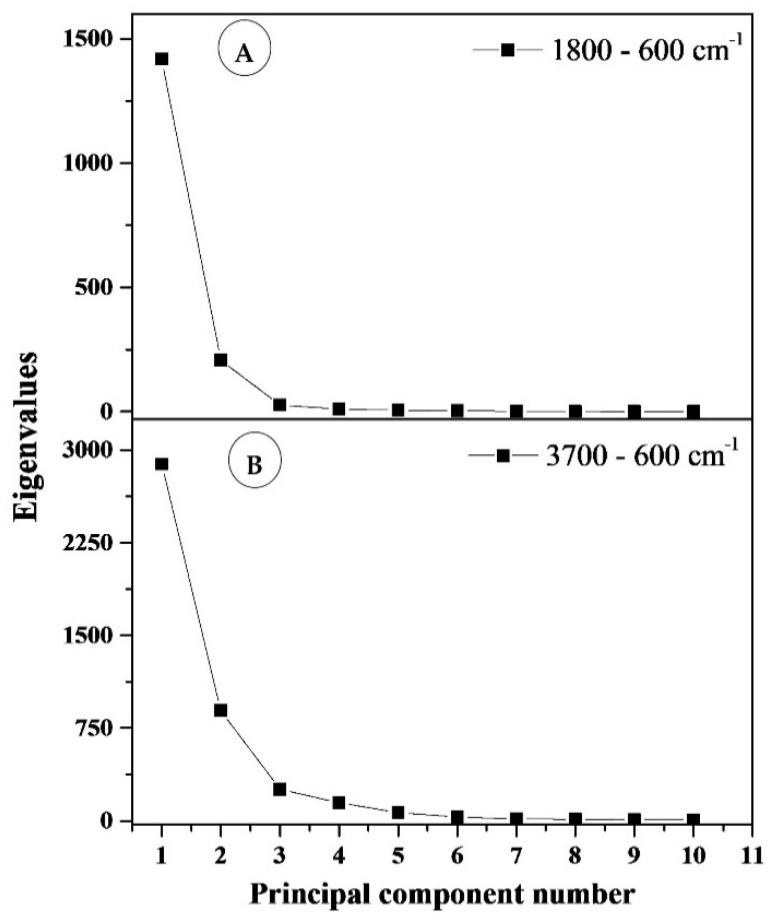
Scree plots (eigenvalues from principal components): (**A**) for the section 1800–600 cm^−1^, (**B**) for the section 3700–600 cm^−1^.

**Figure 7:**
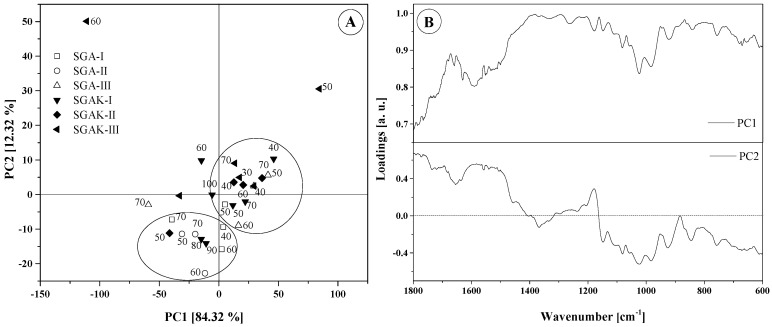
Results of PCA analysis: (**A**) Score plot (numbers on graph are rpm during film blowing); (**B**) Loadings plot for region 1800–600 cm^−1^.

In order to evaluate the main differences in the FTIR spectra of the biodegradable films, multivariate analysis was applied to two regions: 3700–600 cm^−1^ and 1800–600 cm^−1^. The contribution of the principal components is shown in Table 5. The most common features among samples are generally expressed by the first few principal components (PCs). A general criterion for determining the number of PCs is a graphical representation known as a scree plot. This is presented in Figure 6. A scree plot represents the graphic relationship between the eigenvalues in decreasing order against the corresponding PCs. The number of PCs can be determined by locating the point at which the graph shows stabilization in the slope. In our analysis, the first two principal components explain more than 96.67% and 87.09% of the total variance (structure of dependence of primary variables) for wavenumber ranges 1800–600 cm^−1^ and 3700–600 cm^−1^, respectively. Based on these results, for both spectral regions, the two main components underwent further study as they exhibit the strongest influence of total variance. The scores and loading plots for PC1 and PC2 for 1800–600 cm^−1^ region is shown in Figure 7.

### 5.1. 1800–600 cm^−1^

For the fingerprint region, the two principal components compel the placement of samples into two main groups. However, some samples could not be grouped. SGAK-III processed at 50 rpm and SGAK-III processed at 60 rpm during film blowing are separated, as according to Figure 5, the intensity of spectra for these samples differs from the others. This effect could affect the result of the analysis. In the case of SGA groups, which differ in potato starch and polyvinyl alcohol content, PCA analysis also displayed a difference between these groups. SGA-II are negatively correlated with PC1, while SGA-III are mainly positively correlated with PC1, and SGA-I is located in the middle of the graph. Moreover, samples from SGAK-I, SGAK-II, and SGAK-II show similarity between groups and it is more difficult to separate them.

It can be observed that the extrusion process and the extruder screw speed are factors determining the division and clustering into groups. Furthermore, all samples obtained at 40 rpm of film-blowing laboratory line are located in the same cluster.

The type of addition also has a significant impact on the classification of the biodegradable film. The use of polyvinyl alcohol allows the division of the studied samples depending on the amount of employed additive. In the case of simultaneous use of polyvinyl alcohol and keratin, the division of samples into clusters is much more complicated. In Figure 7B, the loading plot reveals the wavenumbers that explain the distinction of samples. The PC1 loading shows that samples that are positively correlated with PC1 in the score plot are characterized by spanning the entire range (1800–600 cm^−1^). The arrangement of points on the PC1 vs. PC2 graph, however, is related to the differences in intensities for the range 1460–760 cm^−1^. The greatest diversity occurs at ~1015 cm^−1^ and ~995 cm^−1^, which is connected with stretching vibrations (C–O) and (C–O–C or C–O–H).

**Figure 8:**
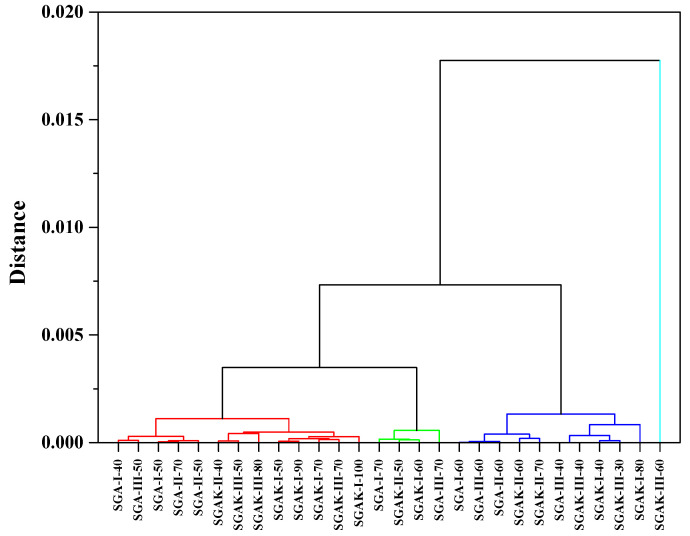
HCA analysis for region 1800–600 cm^−1^. Linkage criteria—Ward method. Clustering metric—Pearson correlation. Different colors indicate similarity clusters.

Figure 8 presents the three diagrams obtained from the hierarchical clustering analysis using the FTIR spectra from the fingerprint region. The application of Ward method linkage and Pearson correlation distance as HCA criteria gives us similar results to that of PCA analysis. The results were divided into four clusters in order to better visualize the data. Here, SGAK-III-60 rpm shows no similarity to other samples.

### 5.2. 3700–600 cm^−1^

**Figure 9:**
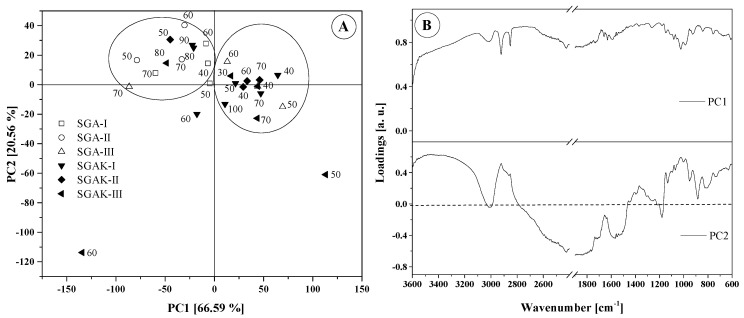
Results of PCA analysis: (**A**) Score plot (numbers on graph are rpm during film blowing); (**B**) Loadings plot for region 3700–600 cm^−1^.

PCA obtained for the 3700–600 cm^−1^ range reveals that the FTIR spectra fell into two different groups, similar to that of the fingerprint region (Figure 9A). Sample SGAK-III processed at 50 rpm and SGAK-III processed at 60 rpm during film blowing are clearly separated from each other. Most samples are, naturally, focused on the center of the graph. Samples from SGA-I and SGA-II are negatively correlated with PC2, whereas SGA-III are positively correlated with PC1. It can be assumed that the contents of potato starch and polyvinyl alcohol have a greater impact on the division of samples rather than the extruder screw speed.

**Figure 10:**
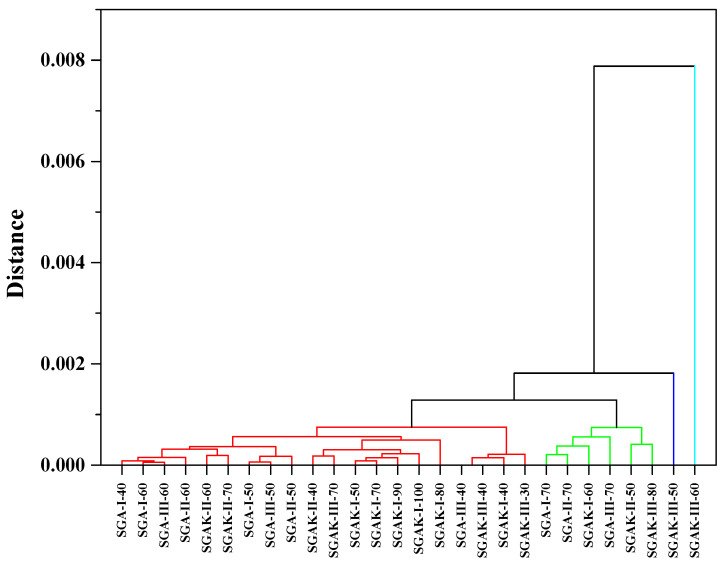
HCA analysis for region 3700–600 cm^−1^. Linkage criteria—average linkage. Clustering metric—Pearson correlation. Different colors indicate similarity clusters.

The loading plot presented in Figure 9B confirms the significant influence of the entire spectral range between 3500–600 cm^−1^ on the arrangements of points in the score plot. From the given range, it follows that samples with the addition of polyvinyl alcohol and polyvinyl alcohol with keratin show significant similarity. In the dendrogram of Figure 10, using average linkage and Pearson correlation as distance measures, we obtain two main clusters. In one, the cluster is more diverse and at 0.001 units of distance can be divided into three clusters, similar to PCA in Figure 9A. Samples SGAK-III processed at 50 rpm and SGAK-III processed at 60 rpm during film blowing showed no similarity to other samples.

In Figure 4 and Figure 5, which present the ATR-FTIR absorption spectra of the produced biodegradable films, and Table 4, which lists the position of the maxima of FTIR absorption spectra, it can be observed that there are small differences in the band shifting. This may be the effect of multivariate analysis. The most significant differences are in the intensity of bands between samples and are due to differences in extruder screw speed.

### 5.3. XRD Results

**Figure 11:**
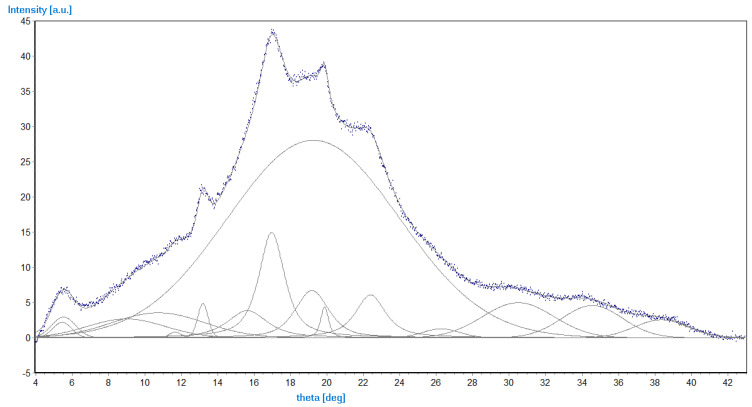
Experimental points (blue) of SGAK-I/50 rpm and model curves (grey). The curve with FWHM (full width at half maximum) of 11° describes the amorphous phase.

Figure 11 presents a typical diffractogram of the investigated polymers. The crystalline phase is characterized by three relatively strong reflections (intensity above 7) at theta of 17.2°, 20.1°, and 22.6°, and three weak ones (intensity 3–4) at 30.5°, 34.6°, and 38.8°. Based on data from Table 6, the average degree of crystallinity for every group of compounds is: SGAK-I 0.378, SGAK-II 0.339, SGAK-III 0.379, SGA-I 0.413, and SGA-III 0.398. The results suggest that the addition of the keratin slightly lowers the content of the crystalline phase. However, the effect of polyvinyl alcohol on the degree of crystallinity is not observed in the investigated range. The amount of crystalline phase also cannot be correlated with the speed of the extruder rotation. This effect can be related to the inhomogeneity of the measured samples, which can be caused by, for example, the different cooling rates of the foil fragments.

## 6. Conclusions

Increase in the extrusion-cooking efficiency was observed upon increase in polyvinyl alcohol and keratin content. Higher values were also obtained for samples containing the two functional additives. In addition, an increase in the amount of polyvinyl alcohol and keratin, results in a lower extrusion-cooking process SME. Furthermore, lower energy consumption values of extrusion-cooking were noted for samples containing both functional additives. It is worth noting that with these processed raw material mixtures, the process was extremely stable and smooth, which is also evidenced by load tests of the plasticizing system in relation to the extruder drive.

Nanoscale studies by means of AFM method application reveal significant differences in biopolymer foil topographical and mechanical characteristics with regard to both sides of the same sample and to the possible effect of keratin. The differences between opposites sides of the same sample are caused by variations in the heat flux through foil/air and foil/holder interface thermal barriers that determine the cooling rate of the melt. In contrast, the effect of keratin is demonstrated in reduction in adhesion forces via the flattening of the sample surface, which might be indirectly related to the changes in specific thermal properties of the material, such as specific heat, softening temperature etc. This is evidenced in that AFM imaging reveals large structural inhomogeneities in cross-sectional views of the biopolymers.

The X-ray diffraction studies also confirm the effect of keratin on tested materials. In this case, the addition of this component lowers the degree of crystallinity, which may reduce the brittleness of the investigated materials. At the same time, no effect of polyvinyl alcohol addition was observed in the investigated range.

The research conducted with the FTIR technique made it possible to assess the effect of the additives on intermolecular interactions in qualitative terms. The research also confirmed the homogeneity of obtained samples, both for SGA and SGAK films, during the preparation of raw material mixtures.

## Figures and Tables

**Table 1:** Raw material mixtures used in the tests.

Sample Code	Potato Starch (%)	Glycerol (%)	Polyvinyl Alcohol (%)	Keratin (%)
SGA-I	79.0	20	1.0	0
SGA-II	78.0	20	2.0	0
SGA-III	77.0	20	3.0	0
SGAK-I	78.0	20	1.0	1.0
SGAK-II	79.0	20	0.5	0.5
SGAK-III	77.0	20	1.5	1.5

SGA—mixture with starch, glycerol, and polyvinyl alcohol addition; SGAK—mixture with starch, glycerol, polyvinyl alcohol, and keratin addition.

**Table 2:** The process efficiency and the specific mechanical energy of the thermoplastic starch extrusion-cooking process with the addition of functional substances.

Parameter	Polyvinyl Alcohol [%]	Keratin [%]	Results
Efficiency (kg h^−1^)	1.0	0	28.9 ± 0.2
	2.0	0	29.6 ± 0.2
	3.0	0	31.2 ± 0.2
	0.5	0.5	29.2 ± 0.2
	1.0	1.0	30.2 ± 0.2
	1.5	1.5	31.8 ± 0.2
SME (kWh kg^−1^)	1.0	0	0.14 ± 0.02
	2.0	0	0.18 ± 0.02
	3.0	0	0.22 ± 0.02
	0.5	0.5	0.18 ± 0.02
	1.0	1.0	0.14 ± 0.02
	1.5	1.5	0.08 ± 0.02

**Table 3:** Results of complex nanoscale mapping of both sides of biopolymer films obtained at 50 rpm of the film-blowing laboratory line.

Sample/Rpm of Film-Blowing Laboratory Line	Film Side	S_q_ [nm]	S_al_ [nm]	S_tr_	Y [MPa]	F_adh_ [nN]
SGA II/50	A	3.71	96.1	0.796	444	1.82
	B	2.82	77.2	0.755	266	2.73
SGA III/50	A	6.25	72.1	0.398	564	2.43
	B	5.44	82.7	0.298	298	2.19
SGAK I/50	A	2.48	68.5	0.132	134	0.967
	B	1.84	78.4	0.505	165	0.809
SGAK II/50	A	3.67	93.2	0.866	925	2.06
	B	2.08	74.9	0.364	333	1.07
SGAK III/50	A	1.36	77.0	0.697	347	1.63
	B	1.18	43.1	0.656	318	1.69

A: internal surface, B: external surface, S_q_: surface roughness (RMS), S_al_: fastest lateral autocorrelation decay, S_tr_: surface texture anisotropy ratio, Y: surface-averaged Young’s pseudo-modulus, F_adh_: surface-averaged tip–surface adhesion force.

**Table 4:** Location of the peaks of FTIR absorption bands, along with the assignment of relevant vibrations to the materials selected for testing biodegradable SGA and SGAK films over the spectral range from 3800 to 500 cm^−1^.

Maximum Position (cm^−1^)		Types and Origin of Vibrations
SGA	SGAK	
3290	3286	ν(–OH) with absorber water or O–H…O–H
2929	2927	ν_as_(C–H)
2878	2886	ν_s_(C–H)
1657 1634	1657 1640	δ_m_(O–H) (e.g., absorber water)
1451 1365	1557 1365	δ(C–H) or δ(CH_2_) in plane
1412	1412	C–H bending and wagging or δ(COH)
1149 1077	1150 1077	anhydroglucose ring C–O stretch of C–O–H in starch and C–O–C antisymmetric bridge
1014 995	1014 995	ν (C–O) and ν(C–O–C or C–O–H)
926 857 761	926 860 731	ν (C–C) and ν (C–O) or C–O–C bend or O–H deformation (broadened by water)

ν-stretching, δ-deformation, s-symmetrical, as-asymmetric, m-medium.

**Table 5:** Eigenvalues, percentage of variance, and cumulative percentage in the data used for the PCA.

Principal Component Number	Eigenvalue	Percentage of Variance (%)	Cumulative (%)
**1800–600 cm^−1^**			
1	1417.510	84.32540	84.32540
2	207.643	12.35235	96.67775
3	27.749	1.65077	98.32852
4	10.786	0.64165	98.97016
5	5.509	0.32773	99.29789
6	3.852	0.22917	99.52706
7	2.880	0.17132	99.69839
8	1.848	0.10996	99.80835
9	0.902	0.05368	99.86202
10	0.630	0.03746	99.89948
**3700–600 cm^−1^**			
1	2888.032	66.52919	66.52919
2	892.380	20.55702	87.08621
3	255.357	5.88246	92.96866
4	146.479	3.37431	96.34297
5	68.177	1.57055	97.91352
6	30.603	0.70497	98.61849
7	14.455	0.33299	98.95148
8	12.699	0.29253	99.24401
9	7.811	0.17992	99.42394
10	6.106	0.14066	99.56460

**Table 6:** Degree of crystallinity of measured samples.

Sample/Rpm of Film-Blowing Laboratory Line	Degree of Crystallinity
SGAK-I/50	0.375
SGAK-I/60	0.381
SGAK-II/50	0.349
SGAK-II/60	0.330
SGAK-III/50	0.377
SGAK-III/60	0.381
SGA-II/50	0.452
SGA-II/60	0.375
SGA-III/50	0.386
SGA-III/60	0.410

## Data Availability

The data presented in this study are available on request from the corresponding authors.
